# Transcriptome-Wide Identification of Cytochrome P450s in Tea Black Tussock Moth (*Dasychira baibarana*) and Candidate Genes Involved in Type-II Sex Pheromone Biosynthesis

**DOI:** 10.3390/insects15020139

**Published:** 2024-02-19

**Authors:** Tiekuang Wang, Xufei Liu, Zongxiu Luo, Xiaoming Cai, Zhaoqun Li, Lei Bian, Chunli Xiu, Zongmao Chen, Qiurong Li, Nanxia Fu

**Affiliations:** 1 Qinghai Academy of Agriculture and Forestry Science, Qinghai University, Xining 810016, China; wang13460060535@163.com; 2 Tea Research Institute, Chinese Academy of Agricultural Sciences, Hangzhou 310008, Chinabianlei@tricaas.com (L.B.);; 3 Key Laboratory of Biology, Genetics and Breeding of Special Economic Animals and Plants, Ministry of Agriculture and Rural Affairs, Hangzhou 310008, China

**Keywords:** tea black tussock moth, Type-II sex pheromone, biosynthesis, cytochrome P450s, RNAseq, phylogenetic analysis

## Abstract

**Simple Summary:**

Sex pheromone plays an important role in mediating mate communication for the tea black tussock moth, *Dasychira baibarana*, which is a devastating pest in Chinese tea plantations. The sex pheromone of this pest is a ternary blend, including two typical Type-II epoxides and one unsaturated ketone. This study investigated P450 candidates that are associated with the biosynthesis of the sex pheromone components. With a combination of comparative RNAseq, phylogenetic, and tissue expression pattern analysis, one CYP4G with abdomen specifically predominant expression profile was likely to be the P450 decarbonylase, and the pheromone-gland specifically and most abundant CYP341B65 was the most promising epoxidase. This research laid a valuable foundation not only for further functional characterization of P450 decarbonylase and epoxidase in the sex pheromone biosynthetic pathway but also for understanding the physiological functions and functional diversity of the CYP gene superfamily in the *D. baibarana*.

**Abstract:**

The tea black tussock moth (*Dasychira baibarana*), a devastating pest in Chinese tea plantations, uses a ternary Type-II pheromone blend containing (3*Z*,6*Z*)-*cis*-9,10-epoxyhenicosa-3,6-diene (*Z*3,*Z*6,epo9-21:H), (3*Z*,6*Z*,11*E*)-*cis*-9,10-epoxyhenicosa-3,6,11-triene (*Z*3,*Z*6,epo9,*E*11-21:H), and (3*Z*,6*Z*)-henicosa-3,6-dien-11-one (*Z*3,*Z*6-21:11-one) for mate communication. To elucidate the P450 candidates associated with the biosynthesis of these sex pheromone components, we sequenced the female *D. baibarana* pheromone gland and the abdomen excluding the pheromone gland. A total of 75 DbP450s were identified. Function annotation suggested six CYPs were orthologous genes that are linked to molting hormone metabolism, and eight antennae specifically and significantly up-regulated CYPs may play roles in odorant processing. Based on a combination of comparative RNAseq, phylogenetic, and tissue expression pattern analysis, one CYP4G with abdomen specifically predominant expression pattern was likely to be the P450 decarbonylase, while the pheromone-gland specifically and most abundant CYP341B65 was the most promising epoxidase candidate for the *D. baibarana* sex pheromone biosynthesis. Collectively, our research laid a valuable basis not only for further functional elucidation of the candidate P450 decarbonylase and epoxidase for the sex pheromone biosynthesis but also for understanding the physiological functions and functional diversity of the CYP gene superfamily in the *D. baibarana*.

## 1. Introduction

The tea black tussock moth, *Dasychira baibarana,* is one of the significant leaf-chewing pests in Chinese tea plantations and has caused considerable economic damage. Additionally, human contact with the larval urticating hairs often results in severe dermatitis [1]. Monitoring and controlling the tea black tussock moth populations with sex pheromones are desirable because sex pheromones are species-specific, nontoxic to other beneficial organisms, and environmentally benign compared to chemical pesticides. And the sex pheromones of this moth was previously identified as a blend of (3*Z*,6*Z*)-*cis*-9,10-epoxyhenicosa-3,6-diene (*Z*3,*Z*6,epo9-21:H), (3*Z*,6*Z*,11*E*)-*cis*-9,10-epoxyhenicosa-3,6,11-triene (*Z*3,*Z*6,epo9,*E*11-21:H), and (3*Z*,6*Z*)-henicosa-3,6-dien-11-one (*Z*3,*Z*6-21:11-one) [2]. However, large-scale application of these compounds for massive control of this pest in tea plantations is hindered by the high cost of mass production with chemical synthesis. Using synthetic biology to produce the tea black tussock moth sex pheromone components is a promising solution [3,4,5]. To explore this solution, the elucidation of the biosynthetic enzymes is essential.

According to the chemical structure, the two components, *Z*3,*Z*6,epo9-21:H and *Z*3,*Z*6,epo9,*E*11-21:H, of the sex pheromones in the tea black tussock moth are typical Type-II sex pheromone, which includes polyene hydrocarbons and their corresponding epoxides [4,6]. While, the unsaturated ketone, *Z*3,*Z*6-21:11-one, belongs to the miscellaneous category [6]. Previous studies have shown Type-II moth sex pheromones with n-3 configuration (3,6,9 double bonds) are derived from dietary linolenic acid (*Z*9,*Z*12,*Z*15-18:COOH) [7]. After being conjugated with CoA ester, they are subjected to successively chain elongation, desaturation (in terms of sex pheromone components with additional double bonds), reduction, and oxidative decarbonylation within the oenocytes, which are associated with either epidermal cells or fat body cells of the abdomen [8,9]. Afterwards, the derived unsaturated hydrocarbons are transported by lipophorin to the pheromone gland for epoxidation [8]. Previous studies have shown that cytochrome P450s (CYPs or P450s) from the CYP4G subfamily, which are highly expressed in the oenocytes, catalyze the decarbonylation for hydrocarbon biosynthesis [10,11]. The P450s enriched specifically in the pheromone gland are required to produce the epoxidized sex pheromone components [9,12,13].

Cytochrome P450s comprise a superfamily of heme-containing monooxygenases that are present in a wide range of organisms, ranging from microorganisms to plants and animals [14]. In insects, CYPs are involved in multiple physiological functions, such as the metabolism of endogenous hormones, adaptation to host plants, and resistance to insecticides [15,16,17].

In the present study, we investigated potential candidate P450s involved in the sex pheromone biosynthesis in the female tea black tussock moth (*D. baibarana*). To achieve this, we initially constructed transcriptome databases for the female pheromone gland and the abdomen excluding the pheromone gland (hereafter referred to as the “abdomen”). Comparative transcriptomic analysis was used for preliminary screening of the P450s that were potentially linked to sex pheromone biosynthesis. The tissue expression profiles of the selected P450s were further assessed using real-time quantitative PCR (RT-qPCR). In addition, the phylogenetic relationships of the CYPs were examined. Our findings served as a foundation for functional elucidation of the vital P450 decarbonylase and epoxidase in the sex pheromone biosynthetic pathway of the tea black tussock moth.

## 2. Results

### 2.1. Transcriptome Sequencing and Functional Annotation of Unigenes

**Figure 1:**
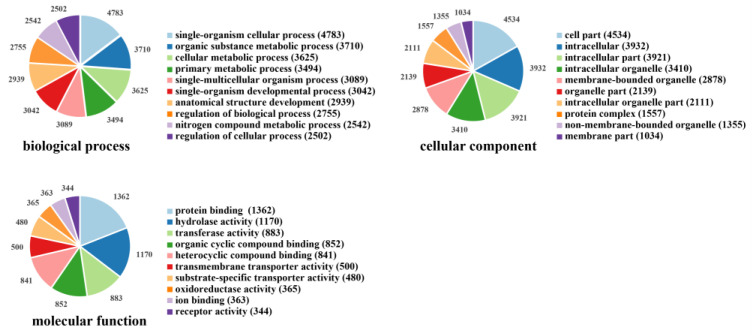
The gene ontology (GO) classification. All unigenes can be classified into one or more categories. The number in each sector represents the total number of unigenes in each category. The analysis was at level 3.

The pheromone gland and abdomen of female tea black tussock moths (*D. baibarana*) were sequenced, generating 34,468 unigenes with an average length of 1143 bp after filtering out the redundant and low-quality sequences. Of these unigenes, 15,219 (44.15%), 7107 (20.62%), 11,624 (33.78%), 7465 (21.66%), and 8941 (25.94%) were annotated in NR, GO, COG, KEGG, and SWSS, respectively (Table 1). These unigenes were classified into three functional groups according to GO annotation, namely biological process, cellular component, and molecular function. As shown in Figure 1, in the biological process classification, the annotated genes were mostly enriched for single-organism cellular processes (4783), followed by organic substance metabolic processes (3710). For cellular component and molecular function categories, these unigenes were predominantly represented in cell parts (4534) and protein binding (1362), respectively. In addition, KEGG analysis revealed that a total of 1081 and 337 unigenes were involved in metabolic pathways and biosynthesis of secondary metabolites, respectively.

### 2.2. Identification of Putative DbCYP Genes

**Figure 2:**
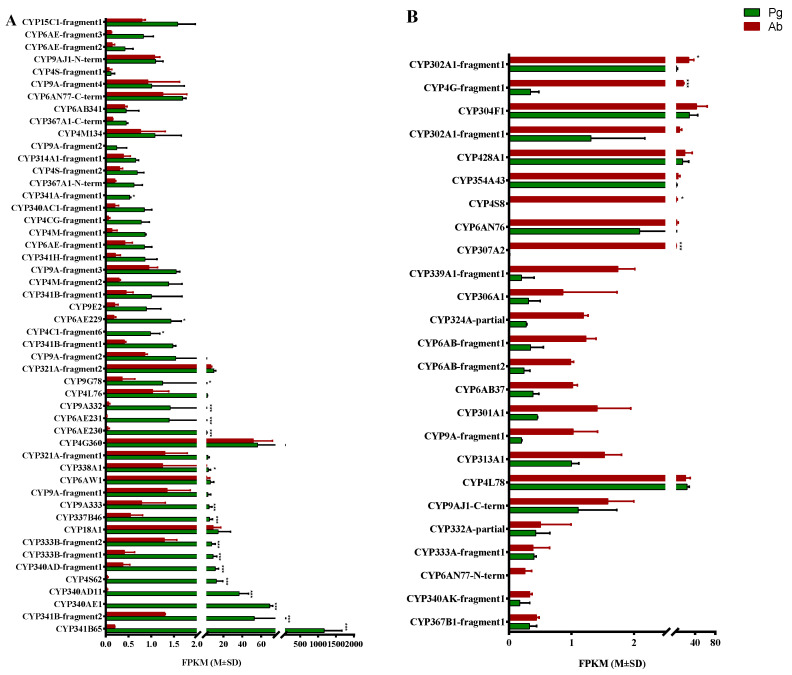
Expression levels of the identified P450s in the tea black tussock moth (*D. baibarana)* based on the FPKM values. (**A**) P450s with higher FPKM values in the pheromone gland; (**B**) P450s with higher FPKM values in the abdomen. Pg and Ab indicated the pheromone gland and the abdomen, respectively. Asterisks represent significant differences between the two tissues based on the Bonferroni *t*-test (* *p* < 0.05, *** *p* < 0.001).

From the black tussock moth pheromone gland and abdomen transcriptomic datasets, a total of 75 CYPs were identified by keyword searching and domain identification. Among them, 25 were with full-length ORF, while the other 50 were partial CYPs. According to the standard nomenclature, these CYPs were categorized into four primary clans: the mitochondrial clan (9 genes), the CYP2 clan (5 genes), the CYP3 clan (33 genes), and the CYP4 clan (28 genes). And the four clans were further classified into 20 families and 36 subfamilies (Table 2). Among them, CYP6 was the largest family (14 CYPs), followed by CYP4 (13 CYPs) and CYP 9 (12 CYPs), see Supplementary File S1. Notably, fifty of these CYPs had higher FPKM values in the pheromone gland than in the abdomen, among which 18 CYPs were statistically significantly more expressed in the pheromone gland (Figure 2A). While the other 25 CYPs exhibited higher FPKM values in the abdomen, and 4 of them were statistically significantly abundant in the abdomen (Figure 2B).

### 2.3. Phylogenetic Analysis

**Figure 3:**
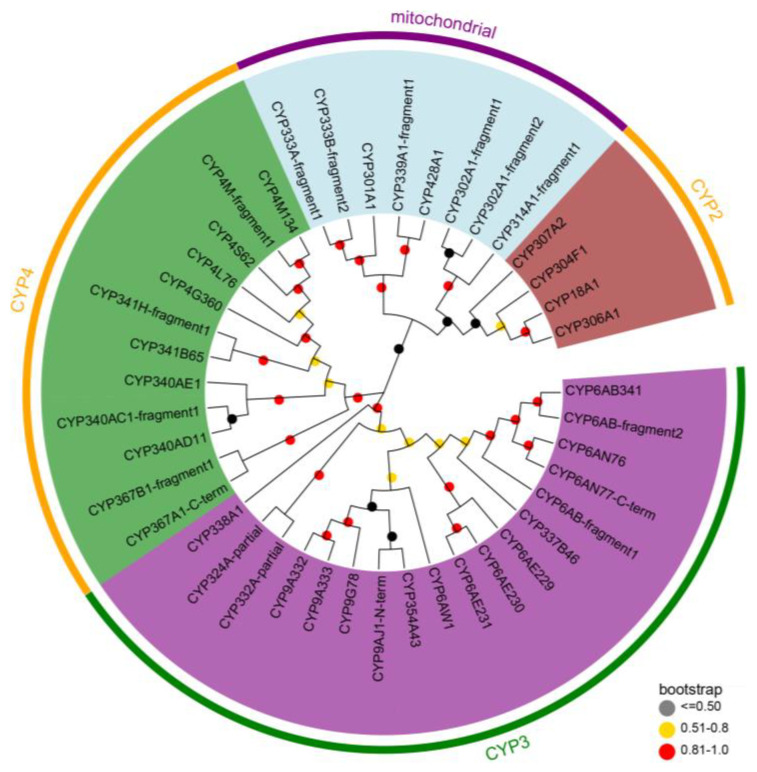
Phylogenetic analysis of *D. baibarana* CYPs. The protein sequences of DbCYPs with more than 100 amino acids were used for tree construction using neighbor-joining method. The bootstrap replicates were 1000. Numbers at each branch point represent the bootstrap values. Different background colors indicated CYP2 clan (brow), CYP3 clan (purple), CYP4 clan (green), and CYP mitochondrial clan (light blue).

To visualize the phylogenetic relationships among the DbCYPs, P450s with more than 100 amino acids were chosen to construct the phylogenetic tree using the neighbor-joining method. As shown in Figure 3, among the 42 DbCYPs, the CYP3 and CYP4 clans possessed more genes and showed a more complex progression of gene expansion, particularly in three families, namely CYP4, CYP6, and CYP9. On the contrary, the CYP2 and mitochondrial clans contained fewer genes and had highly conserved clusters. For example, in the CYP2 clan, three (CYP306A1, CYP307A1, and CYP18A1) of the four CYPs were orthologs of the Halloween genes, which are related to molting hormone, 20-hydroxyecdysone, metabolism. In fact, in the *D. baibarana* transcriptome, a total of five Halloween genes were identified, including one DbCYP307A1, one DbCYP306A1, two DbCYP302A1, and one DbCYP314A1 (Supplementary File S1). In addition, one juvenile hormone biosynthesis-related P450, CYP15C1, was also identified in *D. baibarana* (Supplementary File S1).

**Figure 4:**
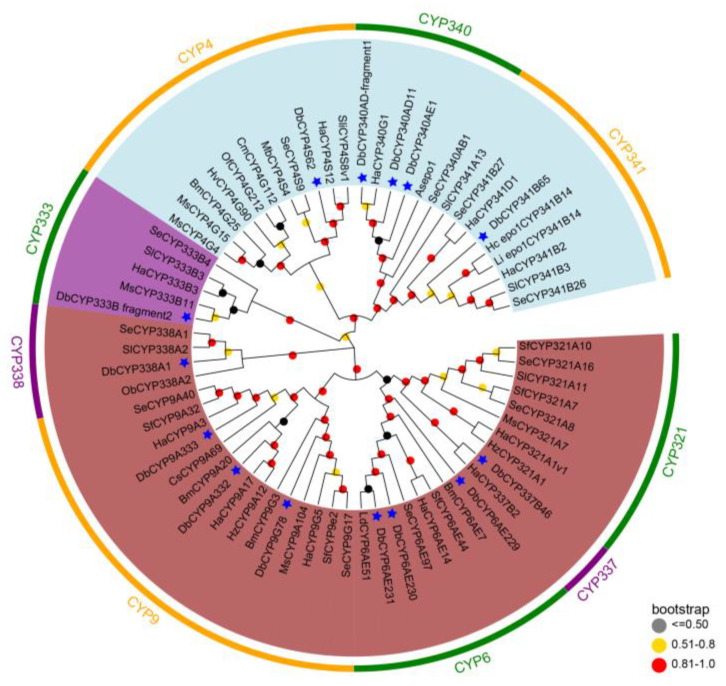
Phylogenetic analysis of *D. baibarana* CYPs expressed predominantly in pheromone glands with those from other insect species. The tree was conducted with MEGA 7.0 based on the amino acid sequences using the maximum likelihood method based on the Poisson model. The bootstrap replicates were 1000. Numbers at each branch point represent the bootstrap values. Different background colors indicated CYP3 clan (brown), CYP4 clan (light blue), and CYP mitochondrial clan (purple). Species abbreviations are as follows: Db, *Dasychira baibarana*; Sl, *Spodoptera litura*; Sf, *Spodoptera frugiperda*; Ha, *Helicoverpa armigera*; Se, *Spodoptera exigua*; Hc, *Hyphantria cunea*; Ms, *Manduca sexta*; Bm, *Bombyx mori*; Ob, *Operophtera brumata*; Hz, *Helicoverpa zea*; Sli, *Spodoptera littoralis*; Mb, *Mamestra brassicae*; Of, *Ostrinia furnacalis*; Cs, Chilo suppressalis; Cm, Cnaphalocrocis medinalis; Ld, Lymantria dispar; Hv, *Heortia vitessoides*; Li, *Lemyra imparilis*; As, *Ascotis selenaria*.

To delineate the phylogenetic relationships among the CYPs expressed predominantly in the pheromone gland of *D. baibarana* with the CYPs from other Lepidopteran species, a phylogenetic tree was constructed. Of the 18 pheromone-gland significantly upregulated CYPs, we selected 14 for analysis. We excluded four CYPs because three of them had less than 100 amino acids while the other one’s FPKM value was below one (Supplementary File S1). As illustrated in Figure 4, the 14 pheromone-gland-specific CYPs were grouped into three distinct clans, namely the CYP3 clan (brown), the CYP4 clan (light blue), and the CYP mitochondrial clan (purple). And the majority were grouped into the CYP3 and CYP4 clan. Since the previously identified epoxidases (Asepo1, Liepo1, and Hcepo1 CYP341B14) belong to the CYP4 clan, the tea black tussock moth epoxidases are supposed to be CYPs within this clan. Specifically, within the CYP4 clan, five CYPs clustered alongside their respective orthologs from other lepidopteran CYPs into three different families. This included three CYPs (CYP340AD11, CYP340AD-fragement1, and CYP340 AE1) in the CYP340 family, one CYP (CYP341B65) in the CYP341 family, and one CYP (CYP4S62) in the CYP4 family. Interestingly, CYP341B65 was aligned within the same clade as the two previously characterized 9,10-epoxidases, CYP341B14.

Additionally, in the CYP3 clan, eight of the pheromone-gland-enriched CYPs were grouped into four different families (CYP6, CYP9, CYP337, and CYP338). And one CYP was clustered with orthologs from the subfamily CYP333B in the mitochondrial clan.

### 2.4. Tissue Expression Profiles of Candidate P450s

**Figure 5:**
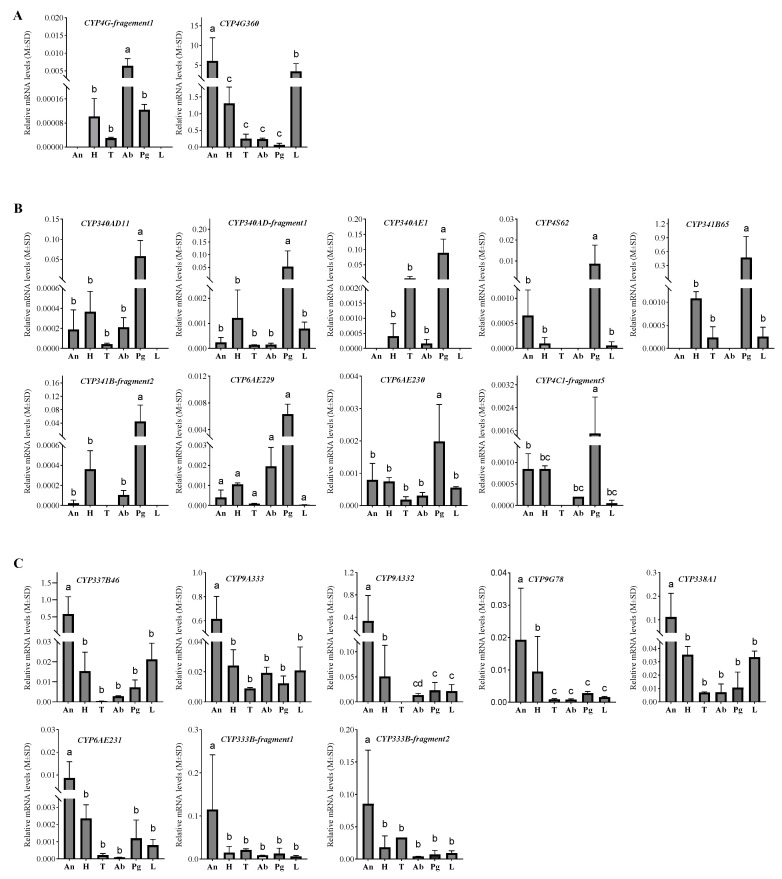
Tissue expressional profiles of the 2 CYP4Gs and 17 pheromone-gland-enriched CYPs in *D. baibarana* based on qPCR analysis. (**A**) expression level of the two CYP4Gs; (**B**) the nine pheromone-gland up-regulated expressed CYPs; (**C**) the eight antennae up-regulated expressed CYPs. Abbreviations: Pg, pheromone gland; Ab, abdomen; An, antennae; H, head; L, leg; T, thorax. Different letters indicated the expression levels are significantly different (*p* < 0.05).

In the *D. baibarana* transcriptome, two DbCYP4Gs were identified. Of them, only CYP4G-fragement1 was up-regulated in the abdomen according to the FPKM value, while CYP4G360 had almost equal expression levels in both the pheromone gland and abdomen (Figure 2). To link their potential role as a decarbonlyase for synthesizing the hydrocarbon precursors of the *D. baibarana* sex pheromones, their expressional profile among different female moth tissues was determined using RT-qPCR analysis. As shown in Figure 5A, CYP4G-fragement1 was found to express with a statistically significant high level in the abdomen compared to the other tissues, including the head, thorax, legs, antennae, and pheromone glands, which is consistent with the RNAseq results (Figure 3). In contrast, CYP4G360 was highly and ubiquitously expressed in all test tissues, with a predominant tendency in the leg and antennae.

The P450s that expressed highly and specifically in the pheromone gland of the female tea black tussock moth are proposed to be the epoxidase candidates. To further narrow down the list of the P450 candidates, the tissue expression profile was used as a screening criterion. Of the 18 CYPs with significantly higher FPKM in the pheromone gland than the abdomen, CYP4C21 was excluded due to its extremely low FPKM value (about 0.5). The tissue-specific expression patterns of the 17 pheromone-gland-enriched CYPs were demonstrated in Figure 5B,C. According to their tissue expression pattern, these CYPs could be divided into two groups, namely the pheromone-gland-enriched and the antennae-enriched groups. As shown in Figure 5B, nine CYPs (CYP340AD11, CYP340AD-fragement1, CYP340AE1, CYP4S62, CYP341B65, CYP341B-fragement2, CYP6AE229, CYP6AE230, and CYP4C-fragement5) exhibited elevated expression levels in the female pheromone gland compared to the other five tissues. Notably, CYP341B65 and CYP340AE1 were the top two most abundantly expressed CYPs in the pheromone gland. And eight CYPs (CYP337B46, CYP9A333, CYP9A332, CYP9G78, CYP338A1, CYP6AE231, CYP333B-fragment1, and CYP333B-fragment2) demonstrated a pronounced expression pattern in the antennae of the female tea black tussock moth, see Figure 5C.

## 3. Discussion

Cytochrome P450s are a superfamily of enzymes found in all kingdoms of life that can catalyze a variety of oxidative transformations of both endogenous and exogenous substrates [18]. In this study, to explore thoroughly the candidate P450s involved in tea black tussock moth (*D. baibarana*) sex pheromone biosynthesis, we constructed the abdomen and pheromone gland transcriptome. A total of 75 P450s were identified. Only a third of them possessed full open reading frames, which is largely due to the low expressional level of these CYPs, as indicated by the low FPKM values shown in the Supplementary File S1. Additionally, the poor assembly of the transcriptomes might also partly account for the observed sequence incompleteness of these CYPs, since no genomic reference information is available for *D. baibarana*. Of all the DbP450s, CYPs in the families of CYP4 and CYP6 accounted for more than half the identified P450s in *D. baibarana*. Cytochrome P450s from the CYP4 and CYP6 families have been reported to be associated with pesticide resistance and host adaptation [19]. Therefore, it is speculated that the apparent CYP expansion in these two families may facilitate the survival of the tea black tussock moth in the tea plantations.

Among these seventy-five DbCYPs, five CYPs (DbCYP307A1, DbCYP306A1, two DbCYP302A1, and DbCYP314A1) were identified as orthologs of the highly conserved Halloween genes. Substantial studies have shown that Halloween genes (CYP307A1/A2, CYP306A1, CYP302A1, CYP315A1, and CYP314A1) are involved in the biosynthesis of the molting hormone, 20-hydroxyecdysone, which plays a key role in regulating the metamorphosis and development of insects [20]. Therefore, it is speculated that these five DbCYPs are likely to play similar roles in the tea black tussock moth (*D. baibarana)*. In addition, we have also identified one ortholog of CYP18A1, and CYP18A1 has been proven to participate in the inactivation of the molting pheromone [21]. Hence, the DbCYP18A1 is supposed to function as the 20-hydroxyecdysone degrading enzyme in *D. baibarana*.

In insects, some CYPs expressed specifically in the antennae play important roles in processing chemical signals, such as the degradation of plant volatiles or perception of pheromones from the external environment. For example, DpCYP345E2, an antennae-specific P450 from the mountain pine beetle, *Dendroctonus ponderosae*, played a role in host plant volatile clearance by oxidizing the pine host monoterpenes [22]. In the tobacco cutworm, *Spodoptera litura*, SlCYP4L4—an antennae predominantly expressed P450—was reported to be involved in the recognition of sex pheromones [23]. In the tea black tussock moth, eight DbCYPs (CYP337B46, CYP9A333, CYP9A332, CYP9G78, CYP338A1, CYP6AE231, CYP333B-fragment1, and CYP333B-fragment2) were expressed specifically at high levels in the antennae compared to the other tissues, suggesting their possible roles in odorant processing.

CYP4G enzymes are insect-specific and highly conserved CYPs. It functions as the oxidative decarbonylase for the synthesis of cuticular hydrocarbons, which serve multiple functions, from desiccation resistance for terrestrial insects to being important signaling molecules for chemical communication [10,24]. To date, most insect genomes carry one (bee and aphid) or two (Drosophila, mosquito, etc.) CYP4G genes [25]. Our results revealed that the tea black tussock moth possesses two CYP4Gs, CYP4G360 and CYP4G-fragement1. Tissue expression profile analysis demonstrated that CYP4G360 was detected to be very highly and ubiquitously expressed in all test tissues, with a predominant tendency in the leg and antennae. Recently, in mosquitoes, *Aedes Aegypti*, a CYP4G35 enriched in the olfactory tissues, was found to play a role in odor processing [26]. It remains unclear whether the newly identified CYP4G360 has similar functions to CYP4G35. In contrast to CYP4G360, CYP4G-fragement1 was significantly accumulated in the abdomen compared to the other tissues, indicating its potential role as oxidative decarbonylases for cuticular hydrocarbon biosynthesis in the oenocytes [11,25]. Interestingly, in the Type-II sex pheromone biosynthetic pathway, the unsaturated hydrocarbon precursors are also synthesized in the oenocytes. Previous studies have demonstrated that the highly conserved CYP4G decarbonylases are nonspecific regarding the chain length of the substrates [10,11,27,28]. Therefore, we proposed that the abdomen-centric CYP4G-fragement1 is also likely to be involved in producing the hydrocarbon sex pheromone precursors, *Z*3,*Z*6,*Z*9-21:H and *Z*3,*Z*6,*Z*9,*E*11-21:H, in the tea black tussock moths. To substantiate this hypothesis, a more comprehensive functional characterization is warranted.

In addition, the preselected pheromone-gland-enriched P450s were examined further to pin out the potential candidate epoxidases in the *D. baibarana* Type-II sex pheromones biosynthetic pathway. Previous studies have shown that CYPs from different families of clan 4 epoxidize region-specifically of the alkenyl precursors in moths, which further shaped the species-specificity of the Type-II sex pheromones. Specifically, As_epo1 (CYP340BD2) from the CYP340 family epoxidized selectively at the *Z*3 double bond, whereas Hc_epo1 (CYP341B14) and Li_epo1 (CYP341B14) from the CYP341 family were the *Z*9 double bond-specific epoxidase [9,12,13]. Therefore, for the tea black tussock moth (*D. baibarana*), it was hypothesized that CYPs from the CYP4 clan with an affinity for the pheromone gland were responsible for the epoxidation of the Z9 double bond of the unsaturated hydrocarbons. With the combination of comparative RNAseq analysis, tissue expression profiles, and phylogenetic analysis, four pheromone-glands specifically enriched CYPs from the CYP4 clan (CYP341B65, CYP340AD-fragment1, CYP340AE1, and CYP340AD11) were found to cluster with the identified epoxidases. Specifically, the pheromone-gland mostly abundant CYP341B65 closely aligned with the two *Z*9 double bond-specific epoxidases, whereas CYP340AE1, CYP340AD11, and CYP340AD-fragment1 were clustered more closer to the *Z*3 double bond-specific epoxidase Asepo1. Collectively, these results suggested that CYP341B65 is likely to play a role in the epoxidation of unsaturated polyene sex pheromone components. Notably, the tea black tussock moth (*D. baibarana*) uses both *Z*3,*Z*6,epo9-21:H and *Z*3,*Z*6,epo9,*E*11-21:H as pheromone components. Previous enzyme assays have demonstrated that although the reported epoxidases could strictly recognize the double bond at the C3 and C9 positions, counting from the C1 position, they exhibited low substrate specificity concerning the aliphatic carbon chain length and the degrees of unsaturation [12,13,29]. Despite this, whether the epoxidases could take *Z*3,*Z*6,*Z*9,*E*11-21:H as substrates remains unknown because previous studies used only monoenes to trienes as substrates. Therefore, it is worth exploring whether the tea black tussock moth utilizes one epoxidase or two independent epoxidases to produce *Z*3,*Z*6,epo9-21:H and *Z*3,*Z*6,epo9,*E*11-21:H. Functional analysis of the above CYP candidates is under the way, and it will ultimately help not only in the identification of the epoxidase(s) for tea black tussock moth sex pheromone biosynthesis but also in the clarification of the epoxidases’ substrate selectivity.

## 4. Materials and Methods

### 4.1. Insect Samples and Tissue Collection

The tea black tussock moth eggs and larvae were collected from Suichang County, Zhejiang Province, China (28.31° N, 119.6° E). Upon emergence, adult male and female moths were fed with a 10% honey water solution separately in 50.5 cm × 50.5 cm × 50.5 cm fine mesh cages. The cages were kept in a controlled climate chamber, maintained at a temperature of 25 ± 1 °C, relative humidity of 70 ± 5%, and a light cycle of 14 L: 10 D. For transcriptome sequencing, the pheromone gland and abdomen of the female moths were dissected 2 days post-eclosion. Fifty pheromone glands were pooled and taken as one biological replicate, while a single abdomen was considered as another separate biological replicate. For RT-qPCR analysis, various tissues, namely the head, thorax, antennae, leg, abdomen, and pheromone gland, were dissected from female moths between 48 and 72 h post-eclosion. For each tissue type sample, three biological replicates were used. All excised tissues were immediately transferred to liquid nitrogen and subsequently stored at –80 °C for later experiments.

### 4.2. cDNA Library Construction and Sequencing

Total RNA was extracted using TRIzol^®^ reagent (Invitrogen, Carlsbad, CA, USA) according to the manufacturer’s instructions. Genomic DNA was removed using DNase I (Takara Bio, Beijing, China). RNA quality was assessed using the Agilent 2100 Bioanalyzer (Agilent, Beijing, China), and quantification was performed with the NanoDrop™ 2000/2000c Spectrophotometers (Thermo Fisher Scientific, Waltham, MA, USA). Approximately 1 μg of high-quality RNA was utilized to construct the cDNA library for Illumina HiSeq sequencing (Illumina Inc., San Diego, CA, USA) using the HiSeq PE150 platform (Mingkebio, Hangzhou, China) in accordance with the manufacturer’s guideline. Detailed methods and analysis of the transcriptome sequencing were performed utilizing the standardized approaches described by Zhang et al. [30].

### 4.3. De Novo Assembly and Annotation

The raw paired-end reads underwent trimming and quality control using Trimmomatic version 0.40 (http://www.usadellab.org/cms/?page=trimmomatic) with the default settings. Trinity (https://github.com/trinityrnaseq/trinityrnaseq) was employed for de novo assembly of the paired-end reads derived from the tea black tussock moth *(D. baibarana)*. The assembled transcripts were then aligned and annotated using BLASTX against various protein databases, namely the nonredundant database (Nr), Clusters of Orthologous Groups (COG), Swiss-Prot (SWSS), and Kyoto Encyclopedia of Genes and Genomes (KEGG), maintaining a typical E-value cutoff of < 1.0 × 10^−5^. The BLASTX outputs from the Nr database were further subjected to GO annotation using Blast2GO (https://www.biobam.com/blast2go/).

### 4.4. Sequence Analysis and Phylogenetic Analysis

Candidate DbCYPs were first selected by keyword search against the annotation results mentioned above. All retrieved sequences were validated using the web-based BLASTX tool available on the NCBI-BLAST network server (http://blast.ncbi.nlm.nih.gov/). After eliminating the redundant candidate sequences, the open reading frames (ORFs) of the remaining candidates were predicted using the ORF finder tool (https://www.ncbi.nlm.nih.gov/orffinder). Subsequently, they were named by the P450 Nomenclature Committee.

To visualize the phylogenetic relationships among the DbCYPs, DbCYPs were aligned using Clustal W implemented in MEGA 7, and a phylogenetic tree was constructed with 1000 bootstrap replicates based on the neighbor-joining method [31]. To further assign putative functions, the pheromone-gland-enriched P450s, together with documented P450 sequences from other insects, were subjected to phylogenetic analysis using the maximum likelihood method based on the Poisson model. Specifically, the CYP dataset contained 14 sequences from *D. baibarana* and 54 from other lepidopteran species. The amino acid sequences used for the phylogenetic analysis of CYPs are provided in the Supplementary File S2.

### 4.5. RT-qPCR Analysis

For the RT-qPCR analysis, 1 μg of total RNA from various tissues (head, thorax, leg, antennae, abdomen, and pheromone gland) was reversely transcribed into cDNA using the HiScript^®^ Q RT SuperMix for qPCR (+gDNA wiper) kit (Vazyme, Nanjing, China). The qPCR procedures were conducted using a Roche LightCycler 480 (Stratagene, La Jolla, CA, USA) in a 20-μL reaction system consisting of 10 µL ChamQ^TM^ SYBR^®^ Master Mix (Vazyme, Nanjing, China), 0.4 µL of each primer, 1 µL of the cDNA template, and 8.2 µL of nuclease-free water. Briefly, after an initial denaturation step at 95 °C for 30 s, the amplifications were carried out with 40 cycles at a melting temperature of 95 °C for 10 s and an annealing/extension temperature of 60 °C for 30 s. Reactions where cDNA was replaced with H_2_O served as negative controls. Each sample had three technical replicates drawn from three biological replicates. Technical replicate with a Cq difference greater than 0.5 was omitted. Glyceraldehyde-3-phosphate dehydrogenase and β-actin were chosen as the reference genes for normalization. Primers were designed using primer3-plus (https://www.primer3plus.com/), and the sequences are documented in the Supplementary File S1. All assays were performed according to MIQE guidelines [32]. Relative gene expression levels were calculated using the comparative 2^−∆∆CT^ method [33].

### 4.6. Statistics

The expression levels of identified transcripts were calculated using the FPKM method (fragments per kilobase of transcript per million mapped reads) using the formula as follows: FPKM (A) = 10^6^ C/(M × L/10^3^) where FPKM (A) represents the expression abundance of a transcript; C (cDNA fragments) is the number of fragments uniquely aligned to a transcript; M (mapped fragments) refers to the total number of fragments uniquely aligned to all identified transcripts; and L stands for the length of transcript (kb). The Bonferroni *t*-test was used to analyze the difference in FPKM between the abdomen and pheromone glands.

To analyze the differences in gene expression levels among multiple tissues, one-way analysis of variance (ANOVA) with Tukey’s post hoc test was performed. The level of significance was set at *p* < 0.05. The figures were constructed with GraphPad Prism version 8.0 (GraphPad Software Inc., La Jolla, CA, USA).

## 5. Conclusions

In the present study, a total of seventy-five P450s were identified in tea black tussock moth, including six CYPs related to molting hormone metabolisms. Phylogenetic relationship coupled with tissue expression profile analysis suggested that the abdomen specifically centric CYP4G-fragement1 is likely to be the decarbonylase, while the pheromone-gland specifically enriched CYP341B65 is the most promising *Z*9 double bond-specific epoxidases for the *D. baibarana* sex pheromone biosynthesis. Our work laid the foundation for further molecular characterization of vital P450 decarbonylase and epoxidase for sex pheromone biosynthesis in the tea black tussock moth (*D. baibarana*) and other lepidopteran insects.

## Figures and Tables

**Table 1:** Summary of the statistics in the tea black tussock moth transcriptome analysis.

Statistics	Data
Total unigenes number	34,468
Total unigenes length	39,402,381
Total transcripts number	59,396
Total transcript length	82,599,491
Average length	1143
Largest unigene	30,524
NR-Annotated unigenes	15,219
GO-Annotated unigenes	7107
COG-Annotated unigenes	11,624
KEGG-Annotated unigenes	7465
SWSS-Annotated unigenes	8941

**Table 2:** Numbers of genes in P450 clans and families identified in the tea black tussock moth (*D. baibarana*).

P450 Clan	CYP2	CYP3	CYP4	Mitochondrial
Total number	5	33	28	9
Family	5	8	5	6
Subfamily	5	13	16	6

## Data Availability

The original data presented in the study are included in the article or the Supplementary Materials; further inquiries can be directed to the corresponding authors.

## Supplementary Materials

The following supporting information can be downloaded at: https://www.mdpi.com/article/10.3390/insects15020139/s1, Supplementary File S1: Information about all identified P450s and primers; Supplementary File S2: Amino acid sequence used to construct the phylogenetic tree in Figure 4.
